# Impact of Elexacaftor-Tezacaftor-Ivacaftor on Gastrointestinal Symptoms, Intestinal Ultrasound, and Pancreatic Stiffness in Cystic Fibrosis

**DOI:** 10.14309/ctg.0000000000000931

**Published:** 2025-10-24

**Authors:** Mirella Fraquelli, Alessandra Piagnani, Fabiola Corti, Chiara Lanfranchi, Giovanni Casazza, Carla Colombo

**Affiliations:** 1 Gastroenterology and Endoscopy Unit, Fondazione IRCCS Ca' Granda, Ospedale Maggiore Policlinico, Milan, Italy;; 2 Cystic Fibrosis Center, Fondazione IRCCS Ca' Granda Ospedale Maggiore Policlinico, Milan, Italy;; 3 Dipartimento di Scienze Cliniche e Comunità, Università degli Studi di Milano, Milan, Italy;; 4 Department of Pathophysiology and Transplantation, Università degli Studi di Milano, Milano, Italy.

**Keywords:** cystic fibrosis, bowel ultrasound, gastrointestinal outcomes, modulator therapy, pancreatic elastography

## Abstract

**INTRODUCTION:**

Elexacaftor-tezacaftor-ivacaftor (ETI) is a highly effective therapy for over 70% of people with cystic fibrosis (pwCF), improving lung disease, quality of life, and survival. The aim of this prospective study was to explore ETI's effects on the gastrointestinal manifestations of cystic fibrosis.

**METHODS:**

In this prospective cross-sectional study, performed in a single tertiary referral center for cystic fibrosis, clinical and laboratory data, intestinal ultrasound (IUS) findings, and pancreatic stiffness (2D-SWE) were assessed at baseline (T0) and during ETI treatment at 6 and 12 months (T6, T12). Abdominal pain, alterations in stool frequency, form, and consistency (diarrhea, constipation) were monitored.

**RESULTS:**

The participants were 86 pwCF (57% male, mean age 21.6 years) and 22 healthy controls enrolled for pancreatic stiffness comparison. IUS abnormalities (e.g., bowel wall thickening, inspissated intestinal contents, lymph node hypertrophy), and abdominal pain (63% at T0 to 2% at T12) significantly decreased (*P* < 0.05). Constipation dropped from 7% at T0 to 0% at T12 and recurrent diarrhea from 77% to 9% (*P* < 0.0001). Pancreatic stiffness normalized after 1-year treatment (T0: 4.21 vs T12: 5.7 kPa, *P* < 0.05). Body mass index increased (T0: 21.0 vs T12: 22.4 kg/m^2^, *P* < 0.001), and glycemic control improved, with reduced fasting glucose (T0: 97.8 vs T12: 86 mg/dL, *P* < 0.001) and hemoglobin A1c (38 vs 36 mmol/mol, *P* < 0.001). High-density lipoproteins cholesterol increased, whereas low density lipoprotein and triglycerides remained stable.

**DISCUSSION:**

ETI normalized IUS parameters and significantly improved pancreatic stiffness, gastrointestinal symptoms, glycemic control, and cholesterol metabolism in pwCF.

## Graphical abstract

**Figure:**
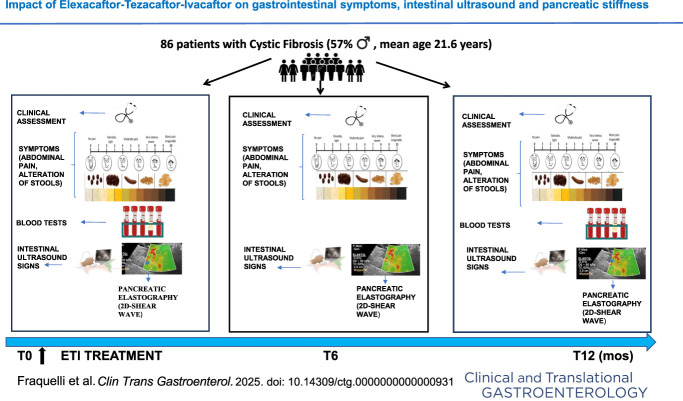

## INTRODUCTION

Mutations in the cystic fibrosis transmembrane conductance regulator (CFTR) gene cause cystic fibrosis (CF), a chronic, autosomal recessive disease characterized by decreased or missing CFTR protein activity which is essential for transepithelial ion transport in exocrine secreting glands (1). At least one-third of the more than 2,000 CFTR sequence variants with various functional implications have been recognized as disease-causing (2). The most prevalent is F508del, which is a deletion of phenylalanine at position 508 of the protein (3).

Lung disease is the major cause of morbidity and mortality in pwCF. However, CF is a multisystemic disease with intestinal, pancreatic, and hepatobiliary involvement of variable severity that may become clinically evident even earlier in life than lung disease.

Pancreatic insufficiency, a direct consequence of CFTR deficiency, is present in at least 70% of pwCF resulting in fat and liposoluble vitamin malabsorption, abdominal symptoms, weight loss, requiring pancreatic enzyme replacement therapy (4). Fecal elastase is the most commonly used test to establish the presence of pancreatic insufficiency (5). Recently, ultrasound elastography has been reported as a promising noninvasive imaging tool for pancreatic evaluation (6); however, its potential role in clinical practice remains to be established. In pwCF lower pancreatic stiffness was documented compared with healthy controls using different elastography techniques (6–9) and attributed to parenchymal adipose replacement because of exocrine pancreatic damage.

At the intestinal level, CFTR dysfunction is involved in the pathogenesis of intestinal obstruction and inflammation. PwCF can develop constipation (10), intestinal obstruction (11,12), mucus accumulation, disturbed motility, dysbiosis with small-bowel bacterial overgrowth (13), and chronic intestinal inflammation. We have previously reported the role of intestinal ultrasound (IUS) for assessing the wide spectrum of intestinal abnormalities in pwCF (14).

Over the last decade, CFTR modulators have been developed to address the basic defect by restoring the CFTR protein function. Clinical trials with the triple combination elexacaftor-tezacaftor-ivacaftor (ETI) have shown beneficial effects on lung disease, quality of life, and survival in pwCF with at least one copy of the F508del mutation (15). However, pulmonary and nutritional outcomes were the primary focus of registration trials, and the impact of modulators on gastrointestinal (GI) symptoms has not yet been well characterized. The aim of this study aim was to assess changes in predefined intestinal parameters assessed by abdominal ultrasound and pancreatic stiffness in pwCF after ETI therapy. Variations of GI symptoms and metabolic laboratory tests were also evaluated.

## METHODS

### Study population

From October 2021 to August 2023, all pwCF aged older than 12 years with at least one F508del mutation, regularly monitored at our CF Center, were prospectively enrolled within 1 month before starting ETI (T0). The gastroenterological assessments were performed by the Division of Gastroenterology of the same Hospital. Only solid organ transplantation was considered an exclusion criterion for enrollment. IUS, pancreatic stiffness measurements, and GI symptoms were assessed at baseline (T0) and after 6 and 12 months (T6 and T12) after ETI start.

Patients were asked for the presence of specific GI symptoms (abdominal discomfort and any changes in the frequency, form, or consistency of stools) that occurred during the 2 weeks before the patient's evaluation. In detail, at least one episode of moderate-to-severe abdominal discomfort (>3 on a 10-point Likert scale), lasting at least 45 minutes, was considered to be indicative of the occurrence of abdominal pain (16). Any alteration of stool frequency and form/consistency was evaluated using the Bristol Stool Scale (17). Constipation was defined by <3 spontaneous bowel movements per week with the presence of hard stool (Bristol stool types 1 or 2) whereas liquid stools (types 6 or 7) were present in case of diarrhea.

At T0 and T12, we collected biochemical data including liver function tests, complete blood count, metabolic profile (including fasting blood glucose, HbA1c, total, high-density lipoproteins (HDL) and low density lipoprotein cholesterol, triglycerides, aspartate aminotransferase and alanine aminotransferase), and plasma fat-soluble vitamin levels. Patients also provided stool samples for fecal elastase-1 levels (FE-1) determination, with exocrine pancreatic insufficiency defined as FE-1 below 200 μg/g feces.

Spirometry was performed at T0 and T12 following current guidelines, and predicted forced expiratory volume in 1 second (ppFEV1) was determined using Global Lung Initiative reference values (18,19). Sweat test was also performed at T0 and T12 by means of pilocarpine iontophoresis.

The study was approved by the Ethics Committee of Milano Area 2 Fondazione IRCCS Ca' Granda–Ospedale Maggiore Policlinico (study number 6401, ID 3009-3010), and all adult patients provided written informed consent. For minors, consent was acquired from parents or guardians along with the child's assent for those aged 12 years and older.

### Intestinal ultrasound

IUS was performed by 2 experienced operators (M.F., A.P.), blinded to clinical and laboratory data using EPIQ Elite device (Philips, Bothell, WA), with a convex low-frequency (3.5–5 MHz) and a linear array high-frequency transducer (5–12 MHz) for the evaluation of the following variables:

1. *Bowel wall thickening*–average of 3 measurements taken from a longitudinal section of both the ileal and colonic tracts (thickened when >3 mm (14)).
2. *Intestinal dilatation*–defined as pathological if >25 mm.
3. *Inspissated intestinal contents (IIC)*–mainly related to increased viscosity and prolonged intestinal transit time, and a typical sign in CF (14).
4. *Intussusception*–the sliding of a part of the intestine into another, represented by ultrasound as a hyperechoic ring in the peripheral and hypoechoic ring at the center (20).
5. *Enlarged mesenteric lymph nodes*–defined as the shortest axis being longer than 5 mm.
6. *Mesenteric adipose tissue hypertrophy*–defined as the presence of a hyperechoic inhomogeneous area surrounding the thickened bowel wall (14).
7. *Increased appendix diameter*–the appendix was considered normal if compressible with a diameter <6 mm.
8. *Free fluid*–presence of free liquid within the peritoneal cavity.

**Figure 1.**
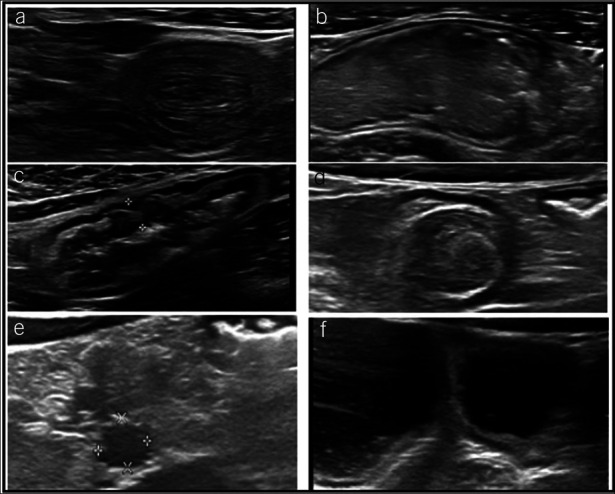
Ultrasonographic findings assessed in 86 patients with CF. (**a**) Small-bowel intussusception as the sliding part of the intestine into an adjacent part of the intestine. (**b**) Inspissated intestinal contents. (**c**) Bowel wall thickened tract (≥3 mm). (**d**) Thickened appendix biparietal diameter (>6 mm). (**e**) Enlarged mesenteric lymph nodes in a hypertrophic mesenteric adipose tissue, defined as a hyperechoic and inhomogeneous area surrounding the thickened bowel wall. (**f**) Intestinal dilatation (>25 mm). CF, cystic fibrosis.

Examples of IUS sign assessed are shown in Figure 1.

### Pancreatic elastography assessment

Pancreatic stiffness was measured using 2D shear-wave elastography (EPIQ Elite; Philips, Bothell, WA) in fasting conditions. The region of interest, with adjustable size, was placed on a portion of the pancreatic parenchyma that could be easily sampled (mainly the head or the body of the pancreas) as previous data showed that the median pancreatic stiffness values for the pancreatic head, body, and tail were superimposable (21). The final value was derived as the median value of 10 measurements (7). The faster the sound beam crossed, the greater the pancreas' stiffness.

A set of 22 consecutive age- and sex-matched healthy controls were compared with the baseline pancreatic elastography readings. Controls were recruited among healthy individuals (healthy siblings of our GI patients, medical and paramedic volunteers) without any GI symptoms.

### Statistical analysis

Kolmogorov-Smirnov test was used to assess the normal distribution of variables, nonparametric tests for non-normal data, and parametric Student *t* test for comparisons between groups and within patients. McNemar test was used to compare paired proportions of patients with GI and IUS alterations and Fisher exact test to evaluate their association at baseline. Analyses were performed in the whole group and in 2 subgroups of patients according to age (<18 vs > 18 years) and F508del genotype (homozygous or heterozygous). Two-sided *P* values of < 0.05 were considered statistically significant. All the statistical analyses were performed with the SAS statistical software (SAS Institute 2023; SAS/STAT 15.3 User's Guide; Cary, NC: SAS Institute).

## RESULTS

We enrolled 86 pwCF whose demographic and clinical characteristics at entry are summarized in Table 1. None of the patients was lost at follow-up. The modifications of IUS findings and GI symptoms from T0 to T6, from T6 to T12, and form T0 to T12 are detailed in Table 2.

**Table 1.** Demographic and clinical characteristics of 86 patients with cystic fibrosis at baseline

Variables	Patients (n = 86)
Male gender, n (%)	49 (57)
Age (yr), mean ± SD	21.6 ± 5.6
BMI (kg/m^2^), mean ± SD	21.0 ± 2.8
F508del homozygous, n (%)	42 (49)
F508del heterozygous, n (%)	44 (51)
FEV1 (% predicted) Sweat chloride (mmol/L)	86.4 ± 19.8 97.4 ± 15.2

Sweat chloride concentrations are considered diagnostic of CF if > 60 mmol/L.

Borderline if 40–60 mmol/L, and normal if <40 mmol/L.

BMI, body mass index; CF, cystic fibrosis; FEV1, forced expiratory volume in 1 second.

**Table 2.** Changes in gastrointestinal symptoms and abdominal ultrasound alterations before (T0) and according to different times (T6 and T12) during treatment with ETI

Variables	Presence	T0	T6	*P* value	T6	T12	*P* value	T0	T12	*P* value
Abdominal pain, n (%)	Yes	54 (62.8)	10 (11.6)	<0.0001	10 (11.8)	2 (2.3)	0.0215	53 (62.3)	2 (2.3)	<0.0001
	No	32 (37.2)	76 (88.4)		75 (88.2)	83 (97.7)		32 (37.7)	83 (9.77)	
Alteration in stool frequency	Yes	72 (83.7)	27 (31.4)	<0.0001	27 (31.8)	8 (9.4)	<0.0001	71 (83.5)	8 (9.4)	<0.0001
and form/consistency	No	14 (16.3)	59 (68.6)		58 (68.2)	77 (90.6)		14 (16.5)	77 (90.6)	
Intestinal intussusception (I)	Yes	39 (45.3)	8 (9.3)	<0.0001	7 (8.2)	1 (1.2)	0.0703	38 (44.7)	1 (1.2)	<0.0001
	No	47 (54.7)	78 (90.7)		78 (91.8)	84 (98.8)		47 (55.3)	84 (98.8)	
Bowel wall thickening (>3 mm)	Yes	27 (31.4)	5 (5.8)	<0.0001	4 (4.7)	3 (3.5)	1.000	26 (30.6)	3 (3.5)	<0.0001
	No	59 (68.6)	81 (94.2)		81 (95.3)	82 (96.5)		59 (69.4)	82 (96.5)	
Intestinal dilatation (>2.5 cm)	Yes	23 (26.7)	6 (7.0)	<0.0001	5 (5.9)	0 (0)	0.0625	22 (25.9)	0	<0.0001
	No	63 (73.3)	80 (93.0)		80 (94.1)	85 (100)		63 (74.1)	85 (100)	
Inspissated intestinal contents	Yes	72 (83.7)	44 (51.2)	<0.0001	43 (50.6)	7 (8.2)	<0.0001	71 (83.5)	7 (8.2)	<0.0001
	No	14 (16.3)	42 (48.8)		42 (49.4)	79 (91.8)		14 (16.5)	78 (9.81)	
MH	Yes	10 (11.6)	4 (4.6)	0.1094	3 (3.5)	0	0.2500	9 (10.6)	0	0.0039
	No	76 (88.4)	82 (95.4)		82 (96.5)	85 (100)		76 (89.4)	85 (100)	
LN	Yes	40 (46.5)	42 (48.8)	0.7055	41 (48.2)	24 (28.2)	0.0023	39 (45.9)	24 (28.2)	0.0059
	No	46 (53.5)	44 (51.2)		44 (51.8)	61 (71.8)		46 (54.1)	61 (71.8)	
FF within the intestinal	Yes	21 (24.4)	17 (19.8)	0.3877	16 (18.8)	4 (4.7)	0.0042	20 (23.5)	4 (4.7)	0.0004
	No	65 (75.6)	69 (80.2)		69 (81.2)	81 (95.3)		65 (76.5)	81 (95.3)	
TA	Yes	40 (46.5)	11 (12.8)	<0.0001	11 (12.9)	1 (1.1)	0.020	39 (45.9)	1 (1.2)	<0.0001
	No	46 (53.5)	75 (87.2)		74 (87.1)	84 (98.9)		46 (54.1)	84 (98.8)	

ETI, elexacaftor-tezacaftor-ivacaftor; FF, free fluid; LN, enlarged mesenteric lymph nodes; MH, mesenteric hypertrophy; TA, thickened appendix.

From baseline to T12, the frequency of abdominal pain significantly diminished from 63% to 2% (*P* < 0.001), and the percentage of patients with alteration in stool frequency and form/consistency decreased from 83.7% to 9.4% (*P* < 0.001). In detail, the frequency of constipation decreased from 7.0% at T0 to 0% at T12 (*P* = 0.0313) and the presence of intermittent diarrhea from 76.7% to 9.3% (*P* < 0.0001).

During the follow-up, none of the patients developed new symptoms apart from 3 patients referring recurrent meteorism. All the predefined IUS signs were significantly less frequent at T12 as compared with T0, and among them, “IICs” was significantly less frequent also at T6 as compared with baseline.

The association between some IUS variables of interest (intestinal intussusception, bowel wall thickening, intestinal dilatation (ID) and IIC) and abdominal pain and alteration of stool frequency and form/consistency at baseline are reported in Table 3. An increased appendix diameter and the presence of intestinal intussusception were significantly related to the presence of abdominal pain, whereas the presence of an inspissated intestinal luminal content was related to alteration in stools frequency and/or consistency.

**Table 3.** Association between the presence of abdominal pain and alteration of stools frequency and/or consistency and IUS findings

Variables	Presence	Abdominal pain	*P* value
Thickened appendix	Yes	38/40 (95.0%)	<0.001
	No	16/46 (34.5%)	
Inspissated intestinal contents	Yes	48/72 (66.6%)	0.130
	No	6/14 (42%)	
Intestinal intussusception	Yes	39/39 (100%)	<0.001
	No	15/47 (31.9%)	
Intestinal dilatation	Yes	18/23 (78.3%)	0.084
	No	36/63 (57.1%)	
Variables	Presence	Alterations of stool frequency and/or consistency	*P* value
Thickened appendix	Yes	35/40 (87%)	0.559
	No	37/46 (80%)	
Inspissated intestinal contents	Yes	72/72 (100%)	<0.001
	No	0/14 (0%)	
Intestinal intussusception	Yes	3/39 (7.7%)	0.077
	No	11/47 (23.4%)	
Intestinal dilatation	Yes	22/23 (95.7%)	0.100
	No	50/63 (79.4%)	

IUS, intestinal ultrasound.

Pancreatic stiffness was assessed in 86 patients and 22 healthy controls (7 healthy siblings of patients with CF and 15 healthy volunteers). Their characteristics were not significantly different from those of the patients. The control group included 11 male patients (50%), the average age was 21.7 ± 5.7 years (14–33), and body mass index (BMI) was 22.6 ± 1.7 (19–26). All of the controls did not report any significant GI symptoms (particularly abdominal pain and changes in stool frequency, shape, or consistency) in the 6 months before to enrollment. In 2 patients pancreatic stiffness measurement was unsuccessful because bowel air interference whereas all measurement were successful among controls.

**Figure 2.**
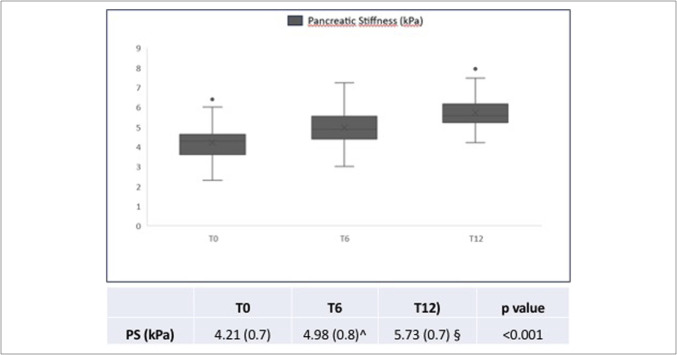
Modification of pancreatic stiffness values in pwCF during ETI therapy. ETI, elexacaftor-tezacaftor-ivacaftor; FE, fecal elastase; pwCF, patients with cystic fibrosis.

At baseline (T0), the mean pancreatic stiffness in pwCF was significantly lower as compared with healthy control (4.21 ± 0.70 kPa vs 6.17 ± 1.12 kPa, *P* < 0.001). Pancreatic stiffness changes during ETI therapy are shown in Figure 2: At T6 and T12, pancreatic stiffness values increased significantly (4.98 ± 0.85 kPa at T6 and 5.73 ± 0.79 at T12, *P* < 0.001) as compared with T0.

FE-1 values were available in 45 of 86 patients (52.3%) at T12. This parameter showed a trend to increase with a median of 203 (interquartile range [IQR] 190–469) at T0 and 438 (IQR 110–500) at T12, without reaching statistical significance (*P* = 0.812), but a clinically relevant improvement (>500 μg/g feces) was observed in 2 patients (16 and 17 years old) after 12 months on ETI therapy. The mean BMI increased from 21 ± 2.8 kg/m^2^ at T0 to 22.2 ± 2.8 kg/m^2^ at T6 (*P* < 0.00 vs T0) and 22.4 ± 2.7 kg/m^2^ at T12 (*P* < 0.001 vs T0).

The biochemical data at baseline and after 1 year of ETI treatment (T12) are summarized in Table 4 and indicated improvement in glycemic control and a significant increase in HDL cholesterol after 1 year of ETI treatment. Serum AST and ALT values remained stable. A significant increase in FEV1 (% of predicted) was observed (from 86.4 ± 19.8 at T0 to 98.7 ± 19.2 at T6, *P* < 0.0001). Sweat chloride values decreased from 97 ± 15 to 50 ± 21 mmol/L at T12 (*P* < 0.0001) and were found to be below the diagnostic threshold for CF in 61 of 86 patients.

**Table 4.** Biochemical data of pwCF at baseline and after 12 months from starting of ETI therapy

	T0 Median (IQR)	T12 Median (IQR)	*P* value^a^
Fasting blood glucose (mg/dL)	93 (83–108)	86 (76–94)	<0.0001
HbA1c (mmol/mol)	38 (35–39)	36 (33–38)	<0.0001
Total cholesterol (mg/dL)	123.5 (103.0–144.0)	124.0 (107.0–143.0)	0.4458
HDL cholesterol (mg/dL)	48.0 (40.0–58.0)	55.0 (43.5–63.0)	<0.0001
LDL cholesterol (mg/dL)	70.0 (54.5–80.0)	67.5 (57.5–79.5)	0.4463
Triglycerides (mg/dL)	87.0 (68.5–108.5)	87.0 (65.0–101.0)	0.2257
AST (U/L)	26.0 (20.0–38.0)	26.0 (20.0–34.0)	0.0977
ALT (U/L)	28.0 (20.0–48.0)	30.0 (21.0–42.0)	0.3300

ETI, elexacaftor-tezacaftor-ivacaftor; HbA1c, glycated hemoglobin; HDL, high-density lipoproteins; IQR, interquartile range; LDL, low density lipoprotein; pwCF, patients with cystic fibrosis.

a Nonparametric test for paired data.

Overall, no statistically significant difference was observed when considering the whole group of patients and the subgroups of patients according to age (<18 vs > 18 years) and genotype (homozygous vs heterozygous for F508del mutation).

## DISCUSSION

The triple-combination ETI includes 2 correctors (Elexacaftor and Tezacaftor) that enhance CFTR trafficking to the cell surface and one potentiator (Ivacaftor) that improves the opening of the CFTR channel already present at the cell surface. In clinical trials and in real-world settings, ETI has demonstrated a remarkable improvement in lung function and nutritional status, as well as a decrease in pulmonary exacerbations (22–24), antibiotic use, and hospitalization (25)^.^ However, the effects on the GI system have not yet been well characterized.

To the best of our knowledge, this is the first study to assess how ETI treatment affects intestinal ultrasonography findings, pancreatic stiffness, and their association with GI manifestations in pwCF. The role of IUS as a noninvasive method for detecting intestinal anomalies in pwCF has already been documented (14,26). In our previous study, pwCF with abdominal symptoms (60% of cases) were those who had more IUS alterations (14).

In this study, abnormal IUS findings were present at enrollment and significantly diminished after 1 year of ETI treatment. Furthermore, the number of participants experiencing abdominal pain and/or alterations in stool frequency or consistency dropped markedly after 6 months of ETI, and this trend was confirmed after 12 months.

At baseline, about one-third of our patients had thickened intestinal wall, increased appendices diameter, and enlarged mesenteric lymph nodes. The chloride transport defect in CF is also evident in the intestinal glands. Slightly thickened gut walls may result from glandular dysfunction in the CF intestine (27). In addition, studies on the appendix and rectum have documented distinctive histological changes, including an increased number of goblet cells and extensively dilated crypts filled with mucus (28).

Noteworthy, before starting, ETI gut dilatation and IICs were present, respectively, in 26% and 83% of our patients, and these findings dramatically declined after 1 year of treatment. We observed a substantial association at baseline between the existence of abdominal pain and the incidence of intussusception and increased appendix diameter. In addition, the presence of IICs was linked to changes in stool frequency and form/consistency, with a borderline significant correlation with intestinal intussusception.

The mechanisms of the effects of ETI on the intestine are probably multiple and may act in different ways (29). Initially, ETI may affect the intestine by improving the activity of the CFTR protein, which in turn improves the movement of fluid and ions across intestinal epithelial cells. Interestingly, magnetic resonance imaging studies reported a lower small-bowel water content in pwCF compared with controls (30); after 24 weeks of ETI therapy, small-bowel water content increased, indicating better intestinal flow at the terminal ileum and better discharge into the colon (31). Restoring CFTR activity at the intestinal level by ETI may therefore improve GI symptoms, lower intestinal inflammation, and secretion quality and another contributing factor may be the reported improvement of intestinal dysbiosis (32).

Consistent with earlier studies (8,^9^,33), we found that at baseline, mean value of pancreatic stiffness in pwCF was considerably lower than that in healthy controls, but during ETI treatment, values increased and approached those of healthy participants. Individuals with similar demographic characteristics who were especially examined for potential symptoms or changes in bowel habits possibly associated with pancreatic insufficiency, hence enabling the approximate exclusion of potential selection bias in comparisons.

This finding may result from pancreatic damage that begins in early childhood with pancreatic atrophy and fatty replacement and subsequently progression to parenchymal fibrosis (34). Although histological changes of the pancreas related to ETI treatment are not yet defined in humans, improved acinar structure, correction of ductal CFTR activity has been shown to reduce ductal obstruction and inflammatory infiltration in animal model of autoimmune disease (35).

Improvement in exocrine pancreatic function in pwCF on CFTR modulator treatment has been documented in clinical trials using FE-1. Modulators may partially restore exocrine pancreatic function, particularly in younger patients by enhancing CFTR function leading to better fluid secretion, reduced mucus plugging, and improved pancreatic microenvironment.

Regarding ETI, in the PROMISE multicenter prospective, observational study, there was no significant change in FE-1 levels in 438 pwCF aged older than 12 years with at least one F508del allele (29) after 6 months of treatment and the increase in our study was, on average, modest. By contrast, most (96.3%) of the 33 patients in the KIWI study (36), a 24-week single-arm study of ivacaftor in children aged 2–5 years with a CFTR gating mutation, had inadequate FE-1 levels at baseline, and by week 24, more than 25% of them displayed an elevation above the clinical cutoff for exocrine pancreatic insufficiency (200 μg/g). Levels of FE-1 observed during KIWI were maintained during an 84 week, open-label extension study; however, no further improvement was observed (37).

Interestingly, in a recent real-word study, age was found to be negatively correlated with change in FE-1, even in the pediatric age (38) However, restoration of exocrine pancreatic function in adult pwCF seems possible (39), especially among those who carry at least 1 functionally milder CFTR mutation.

Most of our patients experienced a significant rise in body weight and BMI since starting ETI, as consistently reported in clinical trials. This finding is likely multifactorial and may be connected to increased bicarbonate secretion, decreased resting energy expenditure, and intestinal inflammation (40).

Regarding laboratory results, after 1 year on ETI, blood levels of HDL cholesterol significantly increased while blood levels of hemoglobin A1c and fasting blood glucose decreased, indicating a positive effect on glucose metabolism (41). One of the study's strengths is that it incorporates imaging (IUS and 2D-SWE), clinical symptoms, and metabolic markers, providing a full perspective of ETI's impact on the GI system. The 12-month follow-up also allowed a temporal evaluation of treatment effects.

Among the study limitations, we could not use a structured questionnaire specifically validated for the assessment of the broad range of GI symptoms in people with CF (16,42), that was not yet available when the study was designed and started. These questionnaires seem to have a higher sensitivity to capture changes brought on by treatment (43) than those validated for other GI pathologies. Instead, we focused on 2 specific symptoms: abdominal pain and alteration in stool form and/or frequency with in-depth qualitative interviews with a recall period of 2 weeks.

A further constraint is the single-center nature of our study that makes its results not generalizable to broader CF populations. Indeed, in the study by Mainz (44) using the CF-Abd-Score (the first validated CF-specific tool for the accurate assessment of CF-related abdominal symptoms), there were marked differences in the response to ETI between the German and UK cohorts, suggesting that several factors may influence GI symptoms in CF including age, severity of pancreatic insufficiency, and dietary factors.

Finally, an adequate CF control group not on ETI treatment could not be included as all patients with the specific genotypes required for ETI prescription were enrolled in this study and could not serve as controls. On the other hand, a control group with different genotypes and clinical expression of the disease could have hampered the causal interpretation of ETI role on GI tract.

In conclusion, improvement of IUS alterations, pancreatic stiffness, GI symptoms, and BMI was documented during ETI treatment for 1 year in pwCF. However, more research is necessary to better understand the complex mechanisms behind the effects of modulator therapy on the GI system.

## CONFLICTS OF INTEREST

**Guarantor of the article:** Mirella Fraquelli MD, PhD.

**Specific author contributions:** M.F., A.P., F.C., C.C.: conception and design of the study. M.F., A.P., F.C., C.L., G.C., C.C.: generation, collection, processing, analysis and/or interpretation of data. M.F., A.P., F.C., C.L., C.C.: drafting and revision of the manuscript. Approval of the final version of the manuscript: All authors.

**Financial support:** This study was part-funded by the Italian Ministry of Health–Current IRCCS research programme.

**Potential competing interests:** None to report.

**Study Highlights**

**WHAT IS KNOWN**

✓ Cystic fibrosis is a chronic, autosomal recessive disease caused by mutations in the cystic fibrosis transmembrane conductance regulator (CFTR) gene, leading to reduced protein activity and increased viscosity of secretions. This results in organ damage, including the lungs, gastrointestinal tract, pancreas, liver, sweat glands, and reproductive systems.
✓ CFTR modulators have been developed to restore protein function, showing benefits in lung disease, quality of life, and survival in patients with F508del mutations.
✓ However, their impact on gastrointestinal symptoms remains uncharacterized.

**WHAT IS NEW HERE**

✓ Our findings showed that elexacaftor-tezacaftor-ivacaftor treatment restored most gastrointestinal parameters in CFTR patients, including body mass index, pancreatic stiffness, gastrointestinal symptoms, and abnormal intestinal ultrasound alterations.
✓ The action mechanisms of elexacaftor-tezacaftor-ivacaftor effects on the gut are likely numerous and complex, and they may act in distinct, sometimes opposing, ways.
✓ More research is needed to better understand the complicated mechanisms that underpin the effects of novel modulator therapies on the gastrointestinal system.

## ABBREVIATIONS:

2D-SWE: 2D shear-wave elastography technique
BMI: body mass index
BWT: bowel wall thickening
CFTR: cystic fibrosis transmembrane conductance regulator
ETI: elexacaftor-tezacaftor-ivacaftor
FE: fecal elastase
FF: free fluid
GI: gastrointestinal
GLI: global lung initiative
HbA1c: glycated hemoglobin
HDL: high-density lipoproteins
I: intussusception
ID: intestinal dilatation
IIC: inspissated intestinal contents
IUS: intestinal ultrasound
LDL: low density lipoprotein
LN: enlarged mesenteric lymph nodes
MH: mesenteric hypertrophy
ppFEV1: predicted forced expiratory volume in one second
pwCF: patients with cystic fibrosis
ROI: region of interest
TA: thickened appendix
